# Nutritional Performance of Grazing Beef Cattle Supplemented with High-Protein Distillers’ Dried Grain

**DOI:** 10.3390/ani14081209

**Published:** 2024-04-17

**Authors:** Milene Rodrigues Dias, Kamila Andreatta Kling de Moraes, André Soares de Oliveira, Erick Darlisson Batista, Ana Maria Rodrigues Salomão, Alexandre Zambenedetti, Natasha Bedresdke Petrenko, Jarliane Nascimento Sousa, Juliana Candeias Ortelam, Alex Ickert, Carla Silva Chaves, Eduardo Henrique Bevitori Kling de Moraes

**Affiliations:** 1 Núcleo de Estudos em Pecuária Intensiva—NEPI, Universidade Federal de Mato Grosso, Campus Universitário de Sinop, Sinop 78550-728, Brazil; md.rodrigues07@gmail.com (M.R.D.); kamilandreatta@gmail.com (K.A.K.d.M.); ranamaria939@gmail.com (A.M.R.S.); alexandrezambenedetti@gmail.com (A.Z.); natashabpetrenko@gmail.com (N.B.P.); jarlianezootecnista@gmail.com (J.N.S.); julianacandeias@outlook.com (J.C.O.); ickertalex4@gmail.com (A.I.); carlazootecnia@gmail.com (C.S.C.); 2 Dairy Cattle Research Lab—NPLEITE, Universidade Federal de Mato Grosso, Campus Universitário de Sinop, Sinop 78550-728, Brazil; andre.oliveira@ufmt.br; 3 Department of Animal Science, Universidade Federal de Lavras, Lavras 37200-900, Brazil; erick.batista@ufla.br

**Keywords:** nitrogen, replacement, rumen undegradable protein, soybean meal

## Abstract

**Simple Summary:**

The strategic supplementation of cattle in an intensive production system seeks to maximize intake and improve tropical forage digestibility, improving production performance per area. In this sense, the use of coproducts, such as distillers grains, becomes an economical alternative due to their lower cost in relation to other traditionally used ingredients. The aim of this study was to evaluate the effects of including high-protein distillers dried grains (HP-DDG; 430 g/CP) in supplements for beef cattle in an intensive grazing finishing system. Five uncastrated male Nellore cattle with an average body w (BW) of 413.5 ± 32 kg were used in a 5 × 5 Latin square design. High-protein distillers’ dried grain (HP-DDG) does not affect the total voluntary intake of beef cattle when replacing soybean meal (SBM). Pasture dry matter intake does not change. Urinary nitrogen excretion decreased with the inclusion of HP-DDG. HP-DDG decreases the proportion of nitrogen compounds that are recycled to the rumen.

**Abstract:**

The objective was to evaluate the effects of including high-protein distillers dried grains (HP-DDG; 430 g/CP) in supplements for beef cattle in an intensive finishing pasture system. Five Nellore bulls with an average body weight (BW) of 413.5 ± 32 kg were distributed in a 5 × 5 Latin square design. The animals were randomly allocated to Marandu palisade grass paddocks (*Urochloa brizantha* cv. Marandu), with 0.32 ha each. Protein-energy supplements were evaluated and formulated with different replacement levels (0, 250, 500, 750 and 1000 g/kg) of soybean meal (SBM) by HP-DDG. Supplements were offered once a day in the amount of 6.0 kg/animal. Replacing SBM with HP-DDG had no effect (*p* > 0.10) on the intake of total and pasture DM, OM, CP, NDFap, digestible organic matter (DOM), metabolizable protein and CP:DOM ratio. Total and pasture DM intake averaged 6.07 and 11.54 kg/day, respectively. Replacing SBM with HP-DDG reduces and increases, respectively, the intake of degradable (RDP) and undegradable (RUP) protein in the rumen (*p* < 0.10) with a consequent linear reduction in ruminal ammonia concentration (RAN), nitrogen excretion in urine and serum N concentration (SUN) (*p* < 0.10). In supplements offered in the amount of 6.0 kg animal/day, SBM can be completely replaced by HP-DDG.

## 1. Introduction

The strategic supplementation of cattle in an intensive production system is undertaken so as to maximize intake and improve forage digestibility, improving production performance per area. In this sense, the use of coproducts, such as distillers’ grains, becomes an economical alternative due to their lower cost in relation to other traditionally used ingredients. Furthermore, dried distillers’ grains (DDG) have a higher portion of rumen undegradable protein (RUP), and once the requirements of the first limiting factor (rumen degradable protein—RDP) are met, the provision of RUP could improve the supply of metabolizable protein (MP) and reduce the proportion of nitrogen compounds that are recycled to the rumen, thus increasing the availability of nitrogen (N) for anabolic purposes [1].

In this way, the use of high-protein DDG (HP-DDG) appears to be an alternative for increasing MP and optimizing the use of N in pasture–supplement systems, thus reducing N excretions [2] and the environmental liabilities generated in the industrial production of ethanol, as well as those related to animal production [3], along with the carbon sequestration carried out by forage plants intended for animal grazing.

Several studies have evaluated the use of wet and dry distillers’ grains dissolved in the diet of production animals [4,5,6,7,8]. However, in most studies, distillers’ grains were evaluated in the diets of cattle in confinement or of dairy cows, and no work has been found evaluating the use of HP-DDG in a production system for beef cattle on pasture.

Research working with HP-DDG for dairy cows showed that there was an increase in true protein and total N in milk and concluded that HP-DDG was effective as a protein supplement for lactating cows [9]. Furthermore, [10] obtained higher feed conversion for lactating cows when HP-DDG was included in the diets.

Therefore, the objective of this study was to evaluate the effects of including HP-DDG in supplements for beef cattle in an intensive finishing system on tropical pasture. Our hypothesis is that HP-DDG can be used as an exclusive protein ingredient for grazing cattle in the transition period between the dry and rainy seasons.

## 2. Material and Methods

The experiment was conducted in Sinop, Mato Grosso, Brazil (11°55’20.89” S, 55°27’33.81” W) during the dry/rainy transition period from August to November 2019. Laboratory procedures were carried out in the laboratory of the Núcleo de Estudos em Pecuária Intensiva—NEPI at the Universidade Federal do Mato Grosso—Sinop Campus. All procedures performed in the experiment were approved by the Research Ethics Committee (REC) of the Universidade Federal do Mato Grosso—UFMT (protocol n°23108.021636/2019-19).

### 2.1. Experimental Area, Design, Animals and Treatments

Before starting the experiment, the area used was idled between the months of April and June 2019, and nitrogen fertilization was carried out with agricultural urea (100 kg of urea/ha; distributed in two applications in the months of March and May/2019). Five Nellore bulls with an average body weight (BW) of 413.5 ± 32 kg were used. The experiment was carried out under a 5 × 5 Latin square design, in which five treatments were tested. Each experimental period consisted of 19 days, with 14 days of adaptation and five days of collection. The animals were randomly distributed in Marandu palisade grass paddocks (*Urochloa brizantha* cv. Marandu), of 0.32 ha each, equipped with individual feeders and drinkers. At the beginning of the experimental period, the animals were weighed and randomly redistributed in the area to minimize the possible effects of the paddocks on the treatments.

Isoprotein protein-energy supplements were evaluated (Table 1) with different replacement levels (0, 250, 500, 750 and 1000 g/kg) of soybean meal (SBM) by HP-DDG (430 g/CP). Supplements were offered once a day, for a total of 6.0 kg per animal at 10:00 in the morning.

### 2.2. Experimental Procedures, Sample Collection and Processing

On the first day of each experimental period, pasture sampling was carried out per paddock to determine the total availability of dry matter (DM) and the chemical composition of the forage. For availability sampling, square metal frames measuring 0.25 m^2^ (0.5 m × 0.5 m) were used. The frames were randomly set up at five points in each paddock, and the total mass of the square was collected 5 cm from the ground. The samples were weighed individually and then homogenized, and a subsample was taken for separation and subsequent determination of the leaf:stem ratio (L:S). After separation, the samples were placed in a forced air ventilation oven at 55 °C for 72 h and weighed again to calculate the total pasture supply and L:S. The total supply of forage in the paddocks averaged 1604.56 ± 720.93 kg DM/ha^,^ and the average L:S was 3.07 ± 1.11 during the experimental period.

To evaluate the chemical composition of the pasture consumed by the animals, grazing simulations were carried out on the first and thirteenth days of the experimental period in all paddocks. The collections were performed manually to capture as closely as possible the way the animals foraged and their preferences, according to the methodology described by [11].

To obtain an estimate of fecal excretion (FE), from the ninth to the sixteenth day, the external marker titanium dioxide (TiO_2_—15 g/animal/day) was applied. Fecal collections were carried out from the fourteenth day of the experimental period for four consecutive days at different times, with the following distribution: 14th day (16:00 h), 15th day (14:00 h), 16th day (12:00 h), and 17th day (08:00 h). Feces was collected after observing the animals’ defecation, taking a contamination-free sample of approximately 300 g/animal. Animals that did not defecate at the scheduled time were sent to the corral for direct collection from the rectum region.

On the seventeenth day of each period, rumen fluid was collected through suction via an esophageal tube four hours after providing the supplements. The samples were filtered, and 50 mL aliquots were fixed with 1.0 mL of sulfuric acid H_2_SO_4_ (1:1), placed in a plastic container and frozen at −20 °C for subsequent determination of the ammonia nitrogen concentration (RAN).

Four hours after providing the supplements, on the last day of the experimental period (19th), blood and urine were collected. Spot urine samples were collected from spontaneous urination or by stimulation of the foreskin. The samples were filtered through a double layer of gauze, and 50 mL of concentrated urine was frozen for the subsequent determination of creatinine and total N concentration. Another 10 mL was diluted in 40 mL of 0.036 N sulfuric acid (H_2_SO_4_) and frozen for subsequent determination of the contents of purine derivatives (PD) (allantoin and uric acid) and urea.

Blood samples were collected by coccygeal puncture using tubes with coagulation accelerating gel (Greiner Bio-One VACUETTE^®^—Vacuette do Brasil, Americana, SP, Brazil) and centrifuged at 4000 rpm for 15 min. The supernatant serum was collected, transferred to a 2 mL Eppendorf tube and frozen at −20 °C for subsequent urea analysis.

### 2.3. Laboratory Analyses

All samples of ingredients, pasture (total sample, leaf, stem and simulated grazing) and feces were dried in a forced air circulation oven at 55 °C for 72 h. To carry out laboratory analysis, the samples underwent particle reduction in sizes of 1 and 2 mm in a Willey knife mill and were stored in duly identified plastic pots. Composite samples of sizes of 1 and 2 mm of feces per animal/treatment/period, and of forage per experimental period (sample/period).

The 1 mm samples were analyzed according to the analytical procedures of [12], regarding the contents of dry matter (DM) (INCT-CA G-001/1 and INCT-CA G-003/1 Methods), mineral matter (MM) (INCT Method -CA M-001/1), total nitrogen (Kjeldahl method), crude protein (CP) (INCT-CA Method N-001/1), neutral detergent insoluble fiber (NDF) (INCT-CA Method F-002/1), neutral detergent insoluble protein (NDIP) (INCT-CA Method N-004/1) and neutral detergent insoluble ash (NDIA) (INCT-CA Method M-002/1). The 2 mm samples were used in in situ incubation analyses to determine indigestible neutral detergent fiber (iNDF) content and effective food degradability (ED) [13].

To determine fecal production, the fecal samples were analyzed for their TiO_2_ concentration using the colorimetry technique described by [14], and its concentration in the samples was related to the daily dose of the indicator (FP = TiO_2_ supplied (g)/fecal TiO_2_ (g/kg fecal DM)).

Samples used for the analysis of neutral detergent fiber corrected for ash and protein (NDFap) were treated with thermostable α-amylase, without the use of sodium sulfite, and the ash and residual protein fractions have been described in [15,16]. To determine the amount of iNDF, an in situ incubation procedure was carried out, and the samples were placed in F57 Ankon^®^ bags and incubated for 288 h [17].

The estimate of voluntary ED intake was quantified based on the relationship between total fecal excretion and its indigestible fraction; for this purpose, NDFi was used as an internal indicator.

$$DMI=\frac{\left[\left(FE\times CIF\right)-IS\right]}{CIFO}+SDMC$$

where DMI = DM intake (g/day); FE = fecal excretion (DM g/day); CIF = concentration of iNDF in feces (g/g); IS = iNDF present in the supplement ingested (kg/day); CIFO = iNDF concentration in forage (kg/kg); and SDMC = supplemented DM intake (kg/day).

The assessment of effective feed degradability was carried out in two rounds, one for concentrated feed and the second for forage assessment (hand plucking). The procedures adopted were carried out in accordance with those described by [13]. Samples of the concentrate and forage, processed to a size of 2 mm, were used for this procedure. Amounts of 2 g was weighed to guarantee a proportion of 20 mg/cm^2^ and placed in Dacron bags 5 cm × 10 cm R510 Ankon^®^ (Macedon, NY, USA), with a porosity of 50 µm. The bags were prepared in triplicate for concentrates and duplicates for forages per food/per animal/per time. The bovine (rumen fistulated) used were adapted to the feed, and subsequently kept under grazing and fed with feed formulated to contain 160 g/kg CP and a concentrate:roughage ratio of 60:40. The bags suspended in the rumen were added to evaluate the degradable fraction at 0, 2, 4, 8, 16, 24, 48 and 72 h for forage, added in reverse order over the incubation time, for simultaneous removal. The time 0 bags were not incubated, but were subjected to the same procedures as the others at the end of the incubation period. The bags were removed from the rumen, washed with constant water changes until clear water was observed, and then were dried in a forced circulation oven at 55 °C for 72 h.

The in situ disappearance kinetics of DM and CP were fitted to the model described by [18], assuming fractional passage rates (kp) of 0.05/h for concentrated feed [19] and 0.039/h for forage [20]. Ruminal DE, rumen degradable protein (RDP) and rumen undegradable protein (RUP) were calculated according to the following equations [19]:

$$RDP=A+B\;\left(\frac{kd}{kd+kp}\right)\times 100$$

$$RUP=B\;\left(\frac{kp}{kd+kp}\right)+C\times 100$$

where A = fraction of CP of complete ruminal degradation (NPN and portion of escape through the pores), B = potentially degraded insoluble protein, with mass degraded in time t determined from kd, kd = rate of degradation, kp = rate of passage, and C = fraction of CP not degraded in the rumen and was estimated as

C=100−(A+B)

Thus, the RDP is composed of the entire fraction A and the portion of fraction B that was really degraded in the rumen. The RUP consists of the fraction of B that escapes degradation in the rumen before being digested, and the entire fraction C. The sum of RDP and RUP is equal to 1000 g/CP.

The RAN was analyzed using Kjeldahl distillation with potassium hydroxide (2 N), according to the technique proposed by [21] and adapted by [22].

Blood serum was analyzed for urea content (serum urea-N (SUN)) using commercial kits (Gold Analisa Diagnóstica Ltd.a, Belo Horizonte, Brazil). Urine samples were analyzed for creatinine, uric acid and urea content using commercial kits (Gold Analisa Diagnóstica Ltd.a). The level of creatinine in urine is constant, so the analysis of this component in samples was used to set a marker in determining urinary volume, according to [23]:

$$CE=\left[0.0345\times \left({RBW}^{0.9491}\right)\right]\times 1000$$

where CE is the equivalent of daily creatinine excretion (mg/BW) and RBW is the reduced body weight (kg).

Allantoin was analyzed in urine samples diluted in H_2_SO_4_ 0.036 N, according to the methodology of [24]. The total excretion of PD (mmol/day) was obtained by summing the concentrations of allantoin and uric acid. This value was used to calculate absorbed purines (AP), according to [24]:

$$AP=\frac{PD-(0.385\times {BW}^{0.75})}{0.85}$$

where 0.385 × BW^0.75^ represents the constant endogenous contribution of PD, and 0.85 is the recovery of AP as PD in urine. The synthesis of ruminal microbial CP (CPmic) was estimated by multiplying the result of the microbial N equation (Nmic) proposed by the N concentration conversion factor [24] (6.25), as follows:

$$CPmic=6.25\times \frac{70\times AP}{\left(0.83\times 0.116\times 1000\right)}$$

where the coefficient 70 amounts to the N content in purines (mg N/mmol), 0.83 assumes 83% digestibility for microbial purines (mmol/d), and 0.116 implies 11.6% purine-N relative to total N in bacteria.

Microbial efficiency (ME) was obtained by dividing g CPmic/digestible organic matter (DOM). Urinary N excretion (UN) was calculated using urinary volume and N concentration in “spot” samples. N retention (NR) was calculated by subtracting UN and fecal N (FN) from total N ingested (Ning).

### 2.4. Statistical Analyses

Data were collected in a 5 × 5 Latin square, with the following effects evaluated: treatment as an experimental fixed effect and animal and experimental period as random effects. We used the MIXED model of [25]

Yijk=μ+Ti+Pj+Ak+εijk

where Yijk is the measured response variable; µ is the general constant; Ti is the fixed effect referring to the treatment (i = 1 to 5); Pj is the random effect referring to the experimental period (j = 1 to 5); Ak is the random effect referring to the animal (k = 1 to 5); and εijk is the residual error.

Data were analyzed by ANOVA at a 10% level of significance. For the response variables with significant effects, comparisons between treatments were carried out using orthogonal polynomials: linear, quadratic, cubic and quartic. Statistical differences between treatments were accepted with a probability ≤ 10%.

## 3. Results

### 3.1. Intake and Digestibility

Replacing SBM with HP-DDG did not affect (*p* > 0.10) the intake of total DM and pasture, OM, CP, NDFap, digestible organic matter (DOM), metabolizable protein or CP:DOM ratio (Table 2). Total DM and pasture intake averaged 6.07 and 11.54 kg/day, respectively. There were, respectively, linear reductions and increases in the intake of degradable protein (RDP) and undegradable protein in the rumen (RUP) (*p* < 0.10), and consequently, a linear reduction in the RDP:DOM ratio due to the replacement of SBM by HP-DDG (Table 2). With the increased inclusion of HP-DDG, no increase in MP intake was observed (*p* > 0.10).

The different levels of HP-DDG had no effect (*p* > 0.10) on the digestibility of DM, OM, CP and NDFap, or the dietary concentration of DOM (Table 3).

### 3.2. Rumen Ammonia Nitrogen, Nitrogen Utilization Efficiency and Microbial Protein Synthesis

With the increase in the level of replacement of SBM by HP-DDG, the concentration of RAM and SUN and the urinary excretion of N decreased linearly (*p* < 0.10). No effects were observed for NR, N use efficiency or ME (Table 4).

## 4. Discussion

The results of the present study unveil the significant potential of the utilization of HP-DDG to replace SBM in protein-energy supplements provided to grazing beef cattle. It is noteworthy that this research is one of the first (or even the first) to be conducted with beef cattle in a pasture–supplements system in Brazil. Nevertheless, further investigation involving a larger sample size of animals is warranted, particularly to comprehensively assess its impact on animal performance.

Our hypothesis that replacing SBM with HP-DDG in the diet of beef cattle would not affect nutritional performance was partially confirmed. Regardless of the replacement level, the inclusion of HP-DDG does not affect the pasture DM intake and other dietary constituents. Similar to our observations, Hubbard et al. [10] found that the addition of 200 g/DM of HP-DDGS did not alter DM intake for dairy cows. Contrary to our findings, [4] reported an increasing trend in DM intake when HP-DDGS (CP = 449 g/kg, NDF = 498 g/and EE = 91.3 g/kg) replaced soybean meal in the diets of dairy cows. Differences between studies in DM intake responses when using DDG in diets may be related to differences in experimental conditions, such as the inclusion rate of soluble corn distillers’ grains, the type of DDG used (HP-DDG, DDGS, WDG, etc.), the types of food ingredients in the basal diet (forage or concentrate, or both) being replaced by distillers’ grains, and the form of feeding (wet or dry) of this byproduct.

One strategy used to reduce dietary protein is to balance the supply of RDP and RUP. [26] developed protein requirement prediction equations for Zebu beef cattle on pasture, according to which the requirements for RDP and RUP would be 746 and 481 g/day, respectively, for animals with an average weight of 400 kg and with an estimated gain of 1.5 kg/day. Therefore, despite there being linear reductions in the RDP and RUP intakes (Table 2) as HP-DDG replaced SBM, it is noted that these values are higher (i.e., 800 and 710 g/day for RDP and RUP, respectively) than the requirement found by [26]; that is, even with the reduction in RDP and RUP intake, HP-DDG provides the minimum requirements to the animals, being able to achieve gains above 1.5 kg/day [26]. These gains are above the national average for pasture animals, which ensures that rearing animals are finished earlier, thus reducing the production cycle.

Using the protein/energy (P:E) ratio appears to be more appropriate for understanding the metabolic effects of protein intake, as it is a more reliable indicator of an animal’s metabolic fitness [27]. Furthermore, this relationship is a recognized parameter that regulates voluntary intake in ruminants [28]. Thus, the maintenance of CP intake was accompanied by the consumption of DOM, which adjusted the synchronism in the CP:MOD ratio of the diet, with a lack of any substitutive effect and enabling the maintenance of pasture consumption. In medium- to high-quality forage situations, providing protein supplements helps maintain the P:E ratio within a comfortable range for the animal, and is compatible with metabolic needs [29].

Nutrient use efficiency can be measured by the relationship between RDP intake and DOM intake at 135 g RDP per kg DOM. In this study, the RDP/DOM ratio reached the optimal standard only in diets where HP-DDGs were not included in the diet (Table 2) and increasing HP-DDG supplementation decreased the RDP/DOM ratio. However, for animals on low- to medium-quality pastures, values of 70 to 110 g of RDP/DOM are recommended.

Supplementation with sources of RUP, such as HP-DDGs, increases the supply of MP, increasing the efficiency of the use of amino acids (AAs). The nutritional value of RUP is determined by the content of essential AA and its proportion and digestibility in MP, also implying the efficiency of protein utilization by ruminants [30]. Thus, the reasons for the lack of responses in the present study may be related to inaccuracies in MP estimation [31] and imbalances in digestible MP and AA, which are often not reported in studies of protein and AA nutrition.

Digestibility is related to the nutritional value of the food presented to the animal; it refers to the ability to use available nutrients to a greater or lesser extent, which is characteristic of the food and not the animal, and thus facilitates the choice of ingredients used [32]. During the extraction of ethanol to obtain DDG, in the drying stage, the digestibility of nutrients can be compromised. However, in the present study, replacing SBM with HP-DDG did not change nutrient digestibility (Table 3). Therefore, HP-DDG can be used to completely replace SBM, since this coproduct showed similar digestibility to SBM.

The rumen nitrogen balance becomes positive for a forage-fed animal when RAN concentrations are greater than 9.7 mg/dL and is maximized when RAN exceeds 15.9 mg/dL [27]. As the RAN concentration represents nitrogen availability in the rumen, a positive association between rumen nitrogen balance and nitrogen utilization efficiency can also be established. Therefore, the efficiency of nitrogen use in the animal’s body is maximized when the RAN concentration exceeds 16.6 mg/dL [27]. Based on these findings, we can establish that replacing soybean meal with HP-DDG up to the level of 750 g maximizes the N balance in the rumen and replacing up to 500 g presents greater N utilization efficiency by the animals, although there was no statistical effect (Table 4).

Previous research suggests that SUN concentrations greater than approximately 5 to 9 mg/dL indicate excessive N intake and N wastage [33,34]. At all inclusion levels used, SUN levels were above 9 mg/dL (Table 4), suggesting that the CP concentration of the diets used may be above the minimum concentration necessary for the animals. However, it is noted that replacing soybean meal with HP-DDG reduced SUN. Gleghorn et al. [35] also determined that increasing the proportion of RUP as a supplemental protein source resulted in lower plasma urea-N concentrations. Furthermore, as RAN decreases, SUN also decreases, as reviewed by [36].

Urinary N excretion is probably a better indicator of N use than SUN because it represents both SUN and animal body weight [37]. The reduction in urinary N excretion as HP-DDG replaced SBM is consistent with the decrease in RAN and suggests that HP-DDG treatments decreased protein degradation in the rumen, leading to decreased ammonia absorption from the rumen and a change in the N excretion route, with proportionally less being excreted in urine (Table 4). Thus, for diets containing excess N, using a high dose of HP-DDG may be beneficial to the environment because the decrease in urinary N excretion would be expected to decrease the volatilization of N in the form of ammonia [38].

Retained nitrogen values (average of 62.28 g/day—Table 4) can be used to estimate the average daily gain in live weight (kg) of the animals [39]. Considering that approximately 750 and 250 g/ kg of meat is water and protein, respectively, and assuming a N to CP ratio of 6.25 (i.e., adopting a body protein N content of 160 g/kg), this implies that the estimated average daily weight gain for steers in both treatments in the present study was approximately 1560 g/day. Assuming an average daily gain of 1560 g/day for animals with an average body weight of 413 kg, the predicted weight gain requirements [26] of the cattle in the present study were approximately 1227 g/day. Therefore, the average dietary CP supply for both treatments was approximately 1490 g/day of CP, which is approximately 21.5% greater than the predicted CP requirements. As a consequence of the excessive amount of CP, there is a greater excretion of N via urine. 

The efficiency of microbial synthesis (EMS) indicates how much energy is directed to nitrogen assimilation by microorganisms [27]. In the present study, the EMS (average 108.4 g MCP/kg DOM), regardless of the replacement level, was below the range proposed in the feeding patterns of 130–170 g MCP/DOM when there is adequate RDP for the ruminal microorganisms [40,41]. However, a higher proportion of grass (i.e., fibrous carbohydrates) in the diet compared to the supplement may have contributed to these similar EMS values.

## 5. Conclusions

It is possible to promote the replacement of up to 1000 g/DM of soybean meal with dry distillers’ grains rich in proteins in protein-energy supplements for cattle on pasture, offered at quantities of up to 6 kg/day, without affecting voluntary intake, or the apparent digestibility of the diet, microbial nitrogen synthesis and nitrogen retention, in addition to reducing N excretion through urine. Further research involving a larger number of animals is recommended, mainly to evaluate the effects on animal performance.

## Figures and Tables

**Table 1:** Proportion of ingredients (g/kg) and chemical composition of supplements and pasture.

Item	Replacement Level (g/kg)					Pasture
	0	250	500	750	1000	
Ingredients (g/kg)						
Mineral mix	15.0	15.0	15.0	15.0	15.0	-
Ground corn	692.0	684.3	676.6	668.8	657.5	-
Soybean meal	293.0	227.5	161.9	96.4	0	-
High-protein dried distillers’ grains	0	73.3	146.5	219.8	327.5	-
Chemical composition (g/kg dry matter)						
Dry matter	907.3	909.4	911.7	914.0	917.3	484.5
organic matter	953.6	955.5	957.9	959.8	963.6	945.5
Crude protein	192.6	194.6	194.6	198.4	199.4	74.6
NDFap ^1^	150.7	158.4	162.8	170.2	179.2	603.6
Rumen degradable protein ^2^	126.7	117.5	106.0	97.5	81.3	49.0
Rumen undegradable protein ^2^	68.9	80.1	91.7	104.0	121.2	26.3
Rumen degradable protein ^3^	671.8	612.2	546.9	489.8	401.6	601.8
Rumen undegradable protein ^3^	328.2	387.8	453.1	510.2	598.4	318.2

^1^ Neutral detergent fiber corrected for ash and protein; ^2^ % dry matter; ^3^ % crude protein.

**Table 2:** Effect of replacing soybean meal with high-protein corn-dried distillers grain on the intake of Nellore bulls at pasture.

Item	Replacement Level (g/kg)					SEM	Treat. (*p* Value) ^5^	Constrast (*p* Value) ^6^	
	0	250	500	750	1000			Linear	Quadratic
	kg/day								
Dry matter	11.7	11.7	11.6	11.7	11.7	0.78	0.457	0.880	0.669
Pasture dry matter	6.1	6.1	6.0	6.1	6.1	0.90	0.679	0.832	0.783
Organic matter	10.9	10.6	10.9	10.5	10.9	0.68	0.689	0.875	0.751
Crude protein	1.5	1.5	1.0	1.5	1.5	0.16	0.688	0.697	0.542
Rumen degradable protein	1.0	0.3	0.9	0.8	0.8	0.17	0.014	0.029	0.725
Rumen undegradable protein	0.4	0.5	0.5	0.6	0.7	0.15	0.046	0.038	0.940
NDFap ^1^	4.7	4.5	4.7	4.4	4.7	0.43	0.741	0.948	0.674
DOM ^2^	7.1	7.1	7.2	7.1	7.2	0.59	0.477	0.442	0.914
Metabolizable protein	0.9	1.0	1.0	1.0	1.0	0.19	0.799	0.557	0.489
CP:DOM ^3^	207	208	210	210	209	10.11	0.812	0.789	0.642
RDP:DOM ^4^	139	130	126	119	112	10.95	0.049	0.068	0.801
	g/body weight								
Dry matter	23.8	23.2	23.3	22.3	23.2	0.16	0.877	0.356	0.582
Pasture dry matter	12.6	11.9	12.1	11.1	11.9	0.26	0.742	0.378	0.607
organic matter	22.6	22.1	22.1	21.3	22.1	0.15	0.754	0.561	0.532
NDFap	9.4	9.0	9.1	8.7	9.2	0.11	0.697	0.515	0.876

^1^ Neutral detergent fiber corrected for ash and protein. ^2^ Digestible organic matter. ^3^ Crude protein:DOM ratio (g/kg). ^4^ Degradable rumen protein:DOM ratio (g/kg). ^5^ Treat (*p*-value) = effect of the treatment evaluated by ANOVA using the F test. ^6^ Contrasts = decomposition of the treatment effect into orthogonal contrasts for linear and quadratic effects. Cubic effect was not presented and was not significant.

**Table 3:** Effect of replacing soybean meal with high-protein corn-dried distillers grain on digestibility and dietary concentration of digestible organic matter of Nellore bulls at pasture.

Item	Replacement Level (g/kg)					SEM	Treat. (*p* Value) ^2^	Constrast (*p* Value) ^3^	
	0	250	500	750	1000			Linear	Quadratic
Dry matter	620	621	623	620	619	1.57	0.741	0.257	0.566
Organic matter	648	653	655	652	642	1.52	0.559	0.112	0.773
Crude protein	744	752	740	752	747	2.16	0.602	0.339	0.404
NDFap ^1^	621	598	605	609	603	1.97	0.998	0.358	0.937
	Dietary concentration (g/kg)								
Digestible organic matter	620	621	622	621	620	13.74	0.502	0.124	0.763

^1^ Neutral detergent fiber corrected for ash and protein. ^2^ Treat (*p*-value) = effect of the treatment evaluated by ANOVA using the F test. ^3^ Contrasts = decomposition of the treatment effect into orthogonal contrasts for linear and quadratic effects. Cubic effect is not presented and was not significant.

**Table 4:** Effect of replacing soybean meal with high-protein corn-dried distillers grain on rumen parameters and N-metabolism of Nellore bulls at pasture.

Item	Replacement Level (g/kg)					SEM	Treat. (*p* Value) ^4^	Constrast (*p* Value) ^5^	
	0	250	500	750	1000			Linear	Quadratic
RAN (mg/dL) ^1^	20.8	19.5	18.6	16.4	15.7	2.04	0.047	0.019	0.785
SUN (mg/dL) ^2^	15.7	14.4	13.5	13.0	12.6	1.49	0.042	0.026	0.977
N-urea urine (g/day)	34.1	38.5	43.3	39.4	43.0	6.59	0.713	0.109	0.441
Nitrogen (g/day)									
Intake	237	237	242	238	240	18.09	0.479	0.648	0.532
Urinary	115	115	113	113	112	7.66	0.019	0.038	0.283
Fecal	62.2	62.0	63.6	62.0	64.0	9.64	0.982	0.593	0.498
Retained	59.1	60.0	64.6	63.7	64.0	11.45	0.777	0.201	0.298
	Efficiency of N utilization (g/g)								
N retained/N intake	0.25	0.25	0.27	0.27	0.27	0.03	0.688	0.492	0.142
N retained/N absorbed	0.26	0.26	0.26	0.26	0.27	0.05	0.723	0.708	0.776
EMS (g MCP/g DOM) ^3^	110	109	106	109	107	16.29	0.459	0.683	0.631

^1^ RAN: rumen ammonia nitrogen. ^2^ SUN: serum urea-N. ^3^ Efficiency of microbial synthesis and MCP: microbial protein. ^4^ Treat (*p*-value) = effect of the treatment evaluated by ANOVA using the F test. ^5^ Contrasts = decomposition of the treatment effect into orthogonal contrasts for linear and quadratic effects. Cubic effect is not presented and was not significant.

## Data Availability

The data presented in this study are available upon request from the corresponding author.
